# A Prognostic Risk Stratification Model to Identify Potential Population Benefiting From Postmastectomy Radiotherapy in T1–2 Breast Cancer With 1–3 Positive Axillary Lymph Nodes

**DOI:** 10.3389/fonc.2021.640268

**Published:** 2021-04-19

**Authors:** Niuniu Hou, Juliang Zhang, Lu Yang, Ying Wu, Zhe Wang, Mingkun Zhang, Li Yang, Guangdong Hou, Jianfeng Wu, Yidi Wang, Bingyao Dong, Lili Guo, Mei Shi, Rui Ling

**Affiliations:** ^1^ Department of Thyroid, Breast and Vascular Surgery, Xijing Hospital, Fourth Military Medical University, Xi’an, China; ^2^ Department of Pathology, Xijing Hospital, Fourth Military Medical University, Xi’an, China; ^3^ Department of Urology, Xijing Hospital, Fourth Military Medical University, Xi’an, China; ^4^ Department of Radiation Oncology, Xijing Hospital, Fourth Military Medical University, Xi’an, China

**Keywords:** breast cancer, lymph nodes, SEER, nomogram, prognosis, postmastectomy radiotherapy

## Abstract

**Background and Objectives:**

To establish a prognostic stratification nomogram for T1–2 breast cancer with 1–3 positive lymph nodes to determine which patients can benefit from postmastectomy radiotherapy (PMRT).

**Methods:**

A population-based study was conducted utilizing data collected from the Surveillance, Epidemiology, and End Results database. Chi-square test or Fisher exact test was used to compare the distribution of characteristics. Cox analysis identified significant prognostic factors for survival. A prognostic stratification model was constructed by R software. Propensity score matching was applied to balance characteristics between PMRT cohort and control cohort. Kaplan-Meier method was performed to evaluate the performance of stratification and the benefits of PMRT in the total population and three risk groups.

**Results:**

The overall performance of the nomogram was good (3-year, 5-year, 10-year AUC were 0.75, 0.72 and 0.67, respectively). The nomogram was performed to excellently distinguish low-risk, moderate-risk, and high-risk groups with 10-year overall survival (OS) of 86.9%, 73.7%, and 62.7%, respectively (P<0.001). In the high-risk group, PMRT can significantly better OS with 10-year all-cause mortality reduced by 6.7% (P = 0.027). However, there was no significant survival difference between PMRT cohort and control cohort in low-risk (P=0.49) and moderate-risk groups (P = 0.35).

**Conclusion:**

The current study developed the first prognostic stratification nomogram for T1–2 breast cancer with 1–3 positive axillary lymph nodes and found that patients in the high-risk group may be easier to benefit from PMRT.

## Introduction

Breast cancer is the most common malignancy, and its mortality rate ranks second among all cancer-related deaths in females (1). Postmastectomy radiotherapy (PMRT) is an effective treatment for breast cancer, which was proposed to reduce local recurrence and prolong survival when rationally combined with systematic treatment (2, 3). PMRT has become an essential therapy for patients of breast cancer with at least four positive lymph nodes.

However, whether PMRT could improve outcomes for T1–2 breast cancer with 1–3 axillary lymph nodes remains controversial. The meta-analysis of the Early Breast Cancer Trialists’ Collaborative Group (EBCTCG) proved that PMRT could effectively decrease the local recurrence of T1–2 breast cancer with 1–3 positive axillary lymph nodes and improve the prognosis (4). Furthermore, the American Society of Clinical Oncology (ASCO) guidelines highly recommend PMRT for T1–2 breast cancer with 1–3 positive axillary lymph nodes (5). But studies have proposed that the 10-year recurrence rate of patients enrolled in clinical trials included in EBCTCG’ meta-analysis was significantly higher compared with T1–2 breast cancer with 1–3 positive axillary lymph nodes in the modern medicine era (20.3% versus 4–10%) (6). Additionally, several retrospective analyses found that PMRT did not decrease the recurrence rate or prolong OS among T1–2 breast cancer with 1–3 positive axillary lymph nodes (7–9). Moreover, the long-term side effects caused by radiotherapies, such as cardiovascular system damage, secondary cancer, and arm lymphedema, could not be ignored (10). These researchers considered that for patients with 1–3 positive axillary lymph nodes, the strong recommendation of PMRT might be unreasonable. Therefore, it is essential for clinicians to screen out the potential population who may benefit from PMRT.

Many studies have tried to identify prognostic factors of T1–2 breast cancer with 1–3 lymph nodes in recent years. Several variables, such as age, tumor size, grade, surgical margin status, prognostic scores, and the number of positive axillary lymph nodes, have been verified as correlative variables with survival (3, 11–24). Additionally, axillary lymph node dissection (ALND) is highly recommended for clinically node-positive breast cancer for its excellent regional control and better prognosis. A prospective single-institution study concluded that the number of positive nodes of ALND and tumor size were both associated with receipt of PMRT (25). Furthermore, several retrospective studies reported that T1–2 breast cancer with 1–3 positive axillary lymph nodes has a great heterogeneity, and the benefit from PMRT would vary significantly in this population (13, 24, 26). Hence, personalized PMRT may benefit the patient to the greatest extent in this heterogeneous population. Nomogram can incorporate all independent prognostic factors, individually predict each patient’s prognosis, and provide a very reliable reference for the treatment (27–29). However, few studies were conducted to establish a prognostic stratification model for T1–2 breast cancer with 1–3 positive axillary lymph nodes.

Based on the data of 11917 patients, the current study aimed to comprehensively analyze prognostic factors of T1–2 breast cancer with 1–3 positive axillary lymph nodes, to establish a prognostic stratification model for patients, and to identify the potential population who could benefit from PMRT.

## Patients and Methods

### Study Population

Data in this study were extracted from the SEER (Surveillance, Epidemiology, and End Results database) 18 Registries, which collects cancer incidence data on patient’s demographics, tumor characteristics, therapy, and follow-up, covers nearly 30% of the population in the United States, and is updated annually. SEER ∗ Stat version 8.3.5 (username:11764-Nov2019) was performed to obtain patients diagnosed with breast cancer in 2000-2014.

The inclusion criteria were listed as follows: (a) female breast cancer was diagnosed as the first and only cancer in 2000-2014 according to the International Classification of Disease, 3rd edition code (ICD-O-3), (b) T1-2, N1, M0 according to the 6th American Joint Committee on Cancer (AJCC), (c) all patients with breast cancer were confirmed by positive histology, (d) ‘malignant’ in behavior code (ICD-O-3). 71008 patients with T1-2, N1 breast cancer were included in our study. Patients were excluded based on the following criteria: (a) incomplete demographic information, such as race and marital status, (b) those younger than 20 or older than 70, (c) bilateral breast cancer and unilateral breast cancer-side unspecified (d) histology types other than infiltrating duct carcinoma (ICD-O-3 code 8500/3) or lobular carcinoma (8520/3), (e) unknown the number of examined lymph nodes, (f) Positive lymph nodes other than 1-3, (g) not received breast mastectomy and underwent axillary surgery (RX Summ–Surg Prim Site: 00, 19, 20, 21, 22, 23, 24) (h) unknown tumor grade, unknown ER status, and PR status, (i) missing PMRT data, (j) incomplete follow-up (survival months = 0).

**Figure 1:**
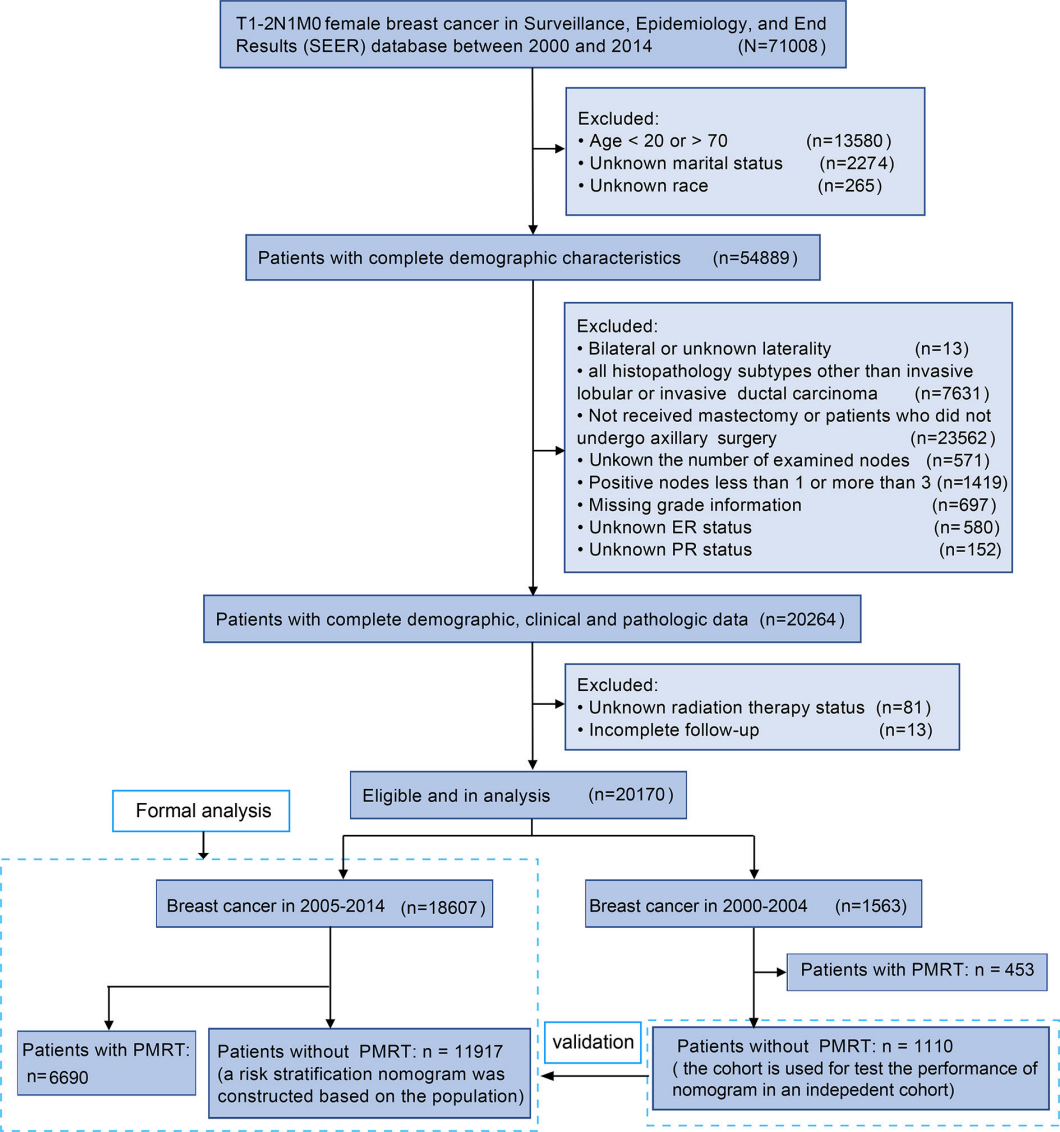
Flowchart of the study design. A total of 20170 female T1–2 breast cancer with 1–3 positive axillary lymph nodes were finally involved in our study.

20170 T1–2 breast cancer with 1–3 positive axillary lymph nodes were finally included in this study. 18607 patients who were diagnosed of T1–2 breast cancer with 1–3 positive axillary lymph nodes between 2005 and 2014 were included for formal analysis. Among them, 6690 (36.0%) patients received PMRT were involved in the PMRT cohort, and 11917 (64.0%) patients did not receive PMRT were involved in the control cohort to construct a nomogram. An independent cohort of 1110 T1-2 breast cancer patients with 1-3 positive nodes in 2000-2004 was selected as the validation cohort. The detailed flowchart for this study was presented in **Figure 1**.

### Covariates

The following clinical and pathological variables were obtained as variables: race, marital status, age at diagnosis, T stage, N stage, histology, grade, ER status, PR status, chemotherapy status, regional nodes examined, regional nodes positive, PMRT status, and follow-up information.

### Outcomes

The main primary endpoint of this study was overall survival (OS) because it was more reliable and objective than recurrence-free survival and could comprehensively reflect the overall benefit of PMRT (the balance between benefit and toxicity of PMRT). OS were defined by survival months and survival status.

### Statistical Analysis

SPSS 22.0 statistical software (IBM Corp., Armonk, NY, USA) was used to analyze the data. The categorical variables were categorized based on previous research results. Chi-square test or Fisher’s exact test was used to compare the baseline characteristics between the PMRT cohort and control cohort, whereas quantitative variables were listed as median with interquartile range (IQR) and compared by Student’s *t* test or non-parametric Mann-Whitney *U* test. The Backward method was used for univariable and multivariable Cox analysis. Variables with P < 0.05 in univariable Cox analysis were incorporated into multivariable Cox analysis to determine independent prognostic factors of the control cohort. The Kaplan-Meier method was performed to calculate the survival rate, and the Log-rank test was applied to compare the differences between the curves.

A backward step-down selection identified a final model according to the Akaike information criterion (30). Based on the multivariable Cox regression results in the control cohort, a nomogram was constructed using R software 3.6.3 (rms package in http://www.r-project.org/) (31). In this study, the bootstrap method = 200 was used to verify the model’s performance. 3-year, 5-year, and 10-year ROC curves and calibration curves were plotted to evaluate the predictive performance of the model (32). The larger the area under the ROC curve (AUC), the higher predictive accuracy of the nomogram. The closer the calibration curve is to the ideal curve, the more unbiased prediction of the model. All T1–2 breast cancer with 1–3 positive axillary lymph nodes were divided into three risk groups (low-risk, moderate-risk, and high-risk group) according to optimal cutoffs of total points of the nomogram constructed in the control cohort. Decision curves were plotted to evaluate the net benefit of the nomogram and nomogram-assisted risk stratification (33). In order to reduce the effect of potential confounding factors on selection bias, the propensity score matching (PSM) without replacement was applied to compare using the nearest-neighbor method with a caliper = 0.02 (34). Standardized mean difference (SMD) was measured for the baseline variable of all independent predictors before and after PSM. SMD of < 0.10 for a given variable demonstrates a relatively small imbalance (35). MatchIt package was used to balance the baseline characteristics between the PMRT cohort and control cohort in different risk groups. By comparing the survival of the PMRT cohort and control cohort in each risk group, we identified a potential population that could benefit from PMRT. The ggplot2 package was performed to plot the Kaplan-Meier curves. Caret package and ggDCA package were applied to draw decision curves. Forestplot package was used to present hazard ratios of PMRT cohort and control cohort in different risk groups.

X-tile was applied to identify the optimal cutoff value of the model scores (36). A two-sided P < 0.05 was considered as statistically significant.

## Results

### Baseline Characteristics in PMRT Cohort and Control Cohort

A total of 18607 T1–2 breast cancer with 1–3 positive axillary lymph nodes in 2005–2014 were finally selected. Among them, 6690 (36.0%) patients received PMRT were involved in the PMRT cohort, and 11917 (64.0%) patients did not receive PMRT were involved in the control cohort to construct a nomogram (**Figure 1**). The median follow-up for the total population was 69 months (IQR, 42–100 months), with 3-year, 5-year, and 10-year OS being 94.7%, 89.7%, and 79.2%, respectively. The median follow-ups for the PMRT cohort and control cohort were 63 months (IQR, 40–93 months) and 73 months (IQR, 44–103 months), respectively. 805 (12.0%) people died in the PMRT cohort, while 1635 (13.7%) people died in the control cohort.

Baseline characteristics between the two cohorts were shown in **Table 1**. Notably, compared with patients in the control cohort, patients in the PMRT cohort were often younger in age, had a significantly higher proportion of Black in race, grade 3, T2, received chemotherapy, performed ALND, fewer examined nodes (≤12), and three positive axillary lymph nodes, and a lower proportion of ER positive and PR positive.

**Table 1:** Demographic and clinicopathologic features of patients between the control cohort and PMRT cohort in T-2 breast cancer with 1-3 positive lymph nodes.

Characteristic	Control cohort (11917)	PMRT cohort (6690)	*P*
Marital status			0.209
Married	7761(65.1)	4418(66.0)	
USDW	4156(34.9)	2272(34.0)	
Age (years)			<0.001
20-39	1223(10.3)	1093(16.3)	
40-49	3465(29.1)	2240(33.5)	
50-59	3908(32.8)	1970(29.4)	
60-69	3321(27.9)	1387(20.7)	
Race			<0.001
White	9333(78.3)	5080(75.9)	
Black	1291(10.8)	845(12.6)	
Other	1293(10.9)	765(11.4)	
Histology			0.967
Infiltrating duct cancer	10871(91.2)	6104(91.2)	
Infiltrating lobular carcinoma	1046(8.8)	586(8.8)	
Grade			<0.001
1	1578(13.2)	582(8.7)	
2	5172(43.4)	2786(41.6)	
3	5167(43.4)	3322(49.7)	
Laterality			0.728
Left	6024(50.5)	3364(50.3)	
Right	5893(49.5)	3326(49.7)	
T stage			<0.001
T1	5346(44.9)	2222(33.2)	
T2	6571(55.1)	4468(66.8)	
Chemotherapy			<0.001
No	3259(27.3)	456(6.8)	
Yes	8658(72.7)	6234(93.2)	
ALND			<0.001
No	4289(36.0)	2074(31.0)	
Yes	7628(64.0)	4616(69.0)	
Examined lymph nodes			0.001
Median (IQR)	12(8-17)	12(7-17)	
Positive lymph nodes			<0.001
1	6701(56.2)	2815(42.1)	
2	3562(29.9)	2198(32.9)	
3	1654(13.9)	1677(25.1)	
ER			<0.001
Negative	2350(19.7)	1490(22.3)	
Positive	9567(80.3)	5200(77.7)	
PR			<0.001
Negative	3526(29.6)	2175(32.5)	
Positive	8391(70.4)	4515(67.5)	

USDW, unmarried/separated/divorced/widowed; Other, American Indian/AK Native, Asian/Pacific Islander; grade 1, well differentiated; grade 2, moderately differentiated; grade 3, poorly differentiated/undifferentiated; ALND, axillary lymph node dissection; ER, estrogen receptor; PR, progesterone receptor; HER2, human epidermal growth factor receptor 2.

### Independent Prognostic Factors in the Control Cohort and Establishment of a Prognostic Nomogram

The 3-year, 5-year, and 10-year OS of 11917 breast cancer in control cohort were 94.6%, 89.6%, and 78.8%, respectively. **Table 2** presented the univariable and multivariable results. Married, other in race, 40-49 in age, grade 1, T1, examined nodes >12, one positive lymph nodes, ER positive, PR positive, and given chemotherapy were independent protective factors for T1–2 breast cancer with 1–3 positive axillary lymph nodes.

**Table 2:** Univariable and multivariable Cox analysis for predicting overall survival in T1–2 breast cancer with 1–3 positive lymph nodes in the control cohort.

Characteristic	Univariable analysis		Multivariable analysis	
	HR(95%CI)	*P*	HR(95%CI)	*P*
Marital Status		<0.001		<0.001
USDW				
Married	0.618(0.561–0.681)		0.687(0.622–0.759)	
Age		<0.001		<0.001
20–39				
40–49	0.740(0.612–0.895)	0.002	0.805(0.666–0.974)	0.026
50–59	0.984(0.821–1.178)	0.858	0.993(0.828–1.190)	0.973
60–69	1.603(1.346–1.909)	<0.001	1.633(1.368–1.949)	<0.001
Race		<0.001		<0.001
White				
Black	1.456(1.270–1.670)	<0.001	1.163(1.011–1.338)	0.035
Other	0.702(0.582–0.847)	<0.001	0.706(0.585–0.852)	<0.001
Histology		0.001		0.132
Infiltrating duct cancer				
Infiltrating Lobular carcinoma	0.728(0.599–0.884)		0.856(0.0.700–1.048)	
Grade		<0.001		<0.001
1				
2	1.379(1.141–1.666)	0.001	1.280(1.058–1.550)	0.011
3	2.268(1.890–2.722)	<0.001	1.632(1.342–1.985)	<0.001
Laterality		0.461		
Left				
Right	1.037(0.941–1.143)			
T stage		<0.001		<0.001
T1				
T2	1.882(1.696–2.088)		1.703(1.532–1.893)	
Chemotherapy		<0.001		<0.001
No				
Yes	0.742(0.669–0.824)		0.718(0.646–0.799)	
ALND		<0.001		0.180
Yes				
No	1.218(1.096-1.355)		1.077(0.966-1.200)	
Examined lymph nodes		0.001		<0.001
4–12				
>12	0.817(0.741–0.901)		0.758(0.687–0.837)	
Positive lymph nodes		<0.001		<0.001
1				
2	1.181(1.056–1.320)	0.258	1.192(1.065–1.334)	0.002
3	1.723(1.517–1.957)	<0.001	1.706(1.499–1.941)	<0.001
ER		<0.001		<0.001
Negative				
Positive	0.492(0.444–0.546)		0.698(0.600–0.812)	
PR		<0.001		<0.001
Negative				
Positive	0.510(0.462–0.562)		0.740(0.643–0.851)	

USDW, unmarried/separated/divorced/widowed; Other, American Indian/AK Native, Asian/Pacific Islander; grade 1, well differentiated; grade 2, moderately differentiated; grade 3, poorly differentiated/undifferentiated; ALND, axillary lymph node dissection; ER, estrogen receptor; PR, progesterone receptor; HER2, human epidermal growth factor receptor 2.

**Figure 2:**
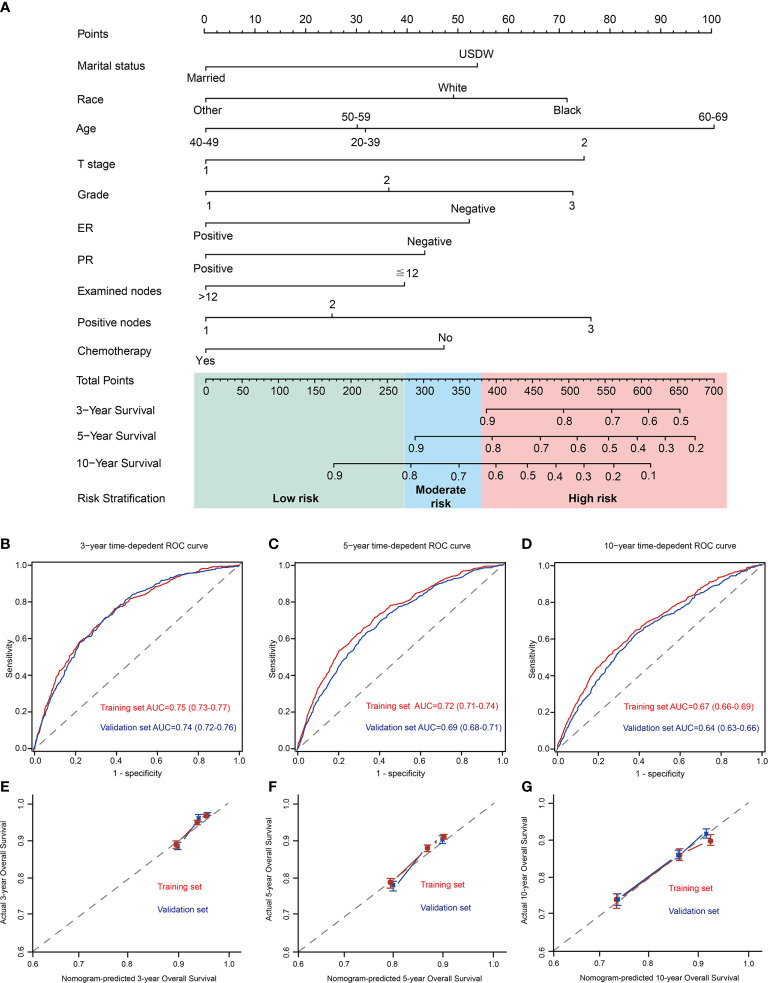
Development of a prognostic stratification nomogram and validation of the proposed nomogram. **(A)** a prognostic stratification nomogram to accurately predict overall survival for T1–2 breast cancer with 1–3 positive lymph nodes. **(B–D)** ROC curves for predicting the overall survival in the internal and external validation at 3-year, 5-year, and 10-year, respectively. The values in brackets of Figure 2B–D represent the area under the ROC curves (AUC). **(E–G)** the calibration curves for predicting patients’ overall survival in the internal and external validation at 3-year, 5-year, and 10-year, respectively. USDW, unmarried/separated/divorced/widowed; Other, American Indian/AK Native, Asian/Pacific Islander; grade 1, well differentiated; grade 2, moderately differentiated; grade 3, poorly differentiated/undifferentiated; ER, estrogen receptor; PR, progesterone receptor.

A nomogram for predicting the OS was created by integrating all independent prognostic factors (**Figure 2A**).

### Validation of the Model’s Performance

In control cohort (internal validation), the prognostic model predicts OS with excellent performance, with 3-year, 5-year, and 10-year AUC were 0.75 (95% CI, 0.73–0.77), 0.72 (95% CI, 0.71–0.74), and 0.67 (95%CI 0.66–0.69), respectively (**Figures 2B–D**). Moreover, the 3-year, 5-year, and 10-year calibration curves further presented excellent agreement between predictions and observation in the probability of 3-year, 5-year, and 10-year survival (**Figures 2E–G**).

An independent cohort of 1110 T1–2 breast cancer patients with 1–3 positive nodes in 2000-2004 were selected as the validation cohort to verify the performance of the nomogram. Baseline characteristics between control cohort and validation cohort were shown in **Supplementary Table 1**. Compared with population in control cohort, patients in validation set have a higher proportion of grade 3, received ALND, infiltrating duct cancer, ER negative and PR negative. In validation cohort (external validation), the prognostic model predicts OS with 3-year, 5-year, and 10-year AUC were 0.74 (95% CI, 0.72–0.76), 0.69 (95% CI, 0.68–0.71), and 0.64 (95%CI 0.63–0.66), respectively (**Figures 2B–D**). Furthermore, the 3-year, 5-year, and 10-year calibration curves further presented high agreement between predictions and observations in the probability of 3-year, 5-year, and 10-year survival in external validation (**Figures 2E–G**).

### Development of Prognostic Stratification Model

**Figure 3:**
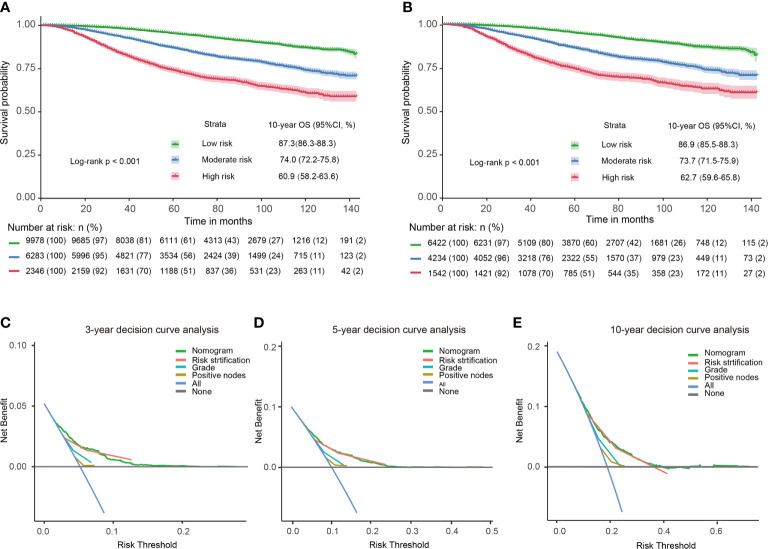
Kaplan-Meier survival curves and decision curves for T1–2 breast cancer with 1–3 positive lymph nodes. Survival curves in the entire cohort before PSM **(A)** and after PSM **(B)** stratified by the total score of the nomogram. 3-year **(C)**, 5-year **(D)**, 10-year **(E)** decision curves show that nomogram and its risk stratification have the highest net benefit almost across the entire threshold probabilities. Blue line: net benefit of a strategy of treating all T1–2 breast cancer with 1–3 positive lymph nodes. Gray line: net benefit of treating no patients of T1–2 breast cancer with 1–3 positive lymph nodes. Colored lines: net benefit of a strategy of treating patients according to the nomogram, risk stratification, grade, and positive nodes.

18607 patients were divided into three prognostic groups based on patients’ total scores using X-tile software (**Supplementary Figure S1**): low-risk group (9978 patients; total score ≤ 274); moderate-risk group (6283 patients; 274 < total score ≤ 380); high-risk group (2346 patients; total score > 380). The survival curves presented excellent discrimination at 10-year OS among the low-risk group, moderate-risk group, and high-risk group, with 10-year OS rates of 87.3%, 74.0%, and 60.9%, respectively (P < 0.001, **Figure 3A**). The baseline characteristics of the PMRT cohort and control cohort in different risk groups were shown in the **Supplementary Table 2**.

PSM was used to balance the independent prognostic factors (marital status, race, age, grade, T stage, examined lymph nodes, positive lymph nodes, ER status, PR status, and chemotherapy status) between the control cohort and PMRT cohort in different prognostic groups. There were 6422, 4234, and 1542 cases T1-2N1M0 breast cancer left in the low-risk group, moderate-risk group, and high-risk group, respectively. As shown in **Table 3**, SMDs of all variables were less than 0.1. The OS rates of the three risk groups after PSM were also significantly different(P < 0.001), with 10-year OS rates of 86.9%, 73.7%, and 62.7%, respectively (P < 0.001, **Figure 3B**). Compared with the low-risk group, hazard ratios (HRs) of the middle-risk group and the high-risk group were 2.465 (95% CI, 2.192–2.772) and 4.449 (95% CI, 3.904–5.070), respectively. The survival curves of breast cancer patients in the three groups were significantly different (P <0.001). Moreover, 3-year, 5-year and 10-year decision curves presented that the net benefit of risk stratification was close to the model’s net benefit and was superior to the net benefit of single factors almost across the entire range of threshold probabilities, further validating that the favorable performance of our risk stratification nomogram (**Figures 3C–E**).

**Table 3:** The baseline characteristics of the patients with PMRT or observation in each risk group based on the PSM.

Characteristic	Low-risk group			Moderate-risk group			High-risk group		
	Control cohort(3211)	PMRT cohort(3211)	SMD	Control cohort(2117)	PMRT cohort(2117)	SMD	Control cohort(771)	PMRT cohort(771)	SMD
Marital Status			0.009			0.011			-0.003
Married	2559(79.7)	2584(80.5)		1211(57.2)	1209(57.1)		265(34.4)	261(33.9)	
USDW	652(20.3)	627(19.5)		906(42.8)	908(42.9)		506(65.6)	510(66.1)	
Age (years)			-0.005			-0.016			-0.021
20-39	453(14.1)	485(15.1)		300(14.2)	332(15.7)		82(10.6)	77(10.0)	
40-49	1499(46.7)	1437(44.8)		511(24.1)	475(22.4)		57(7.4)	83(10.8)	
50-59	995(31.0)	1028(32.0)		647(30.6)	644(30.4)		218(28.3)	196(25.4)	
60-69	264(8.2)	261(8.1)		659(31.1)	666(31.5)		414(53.7)	415(53.8)	
Race			-0.006			-0.001			0.027
White	2519(78.4)	2466(76.8)		1649(77.9)	1641(77.5)		549(71.2)	526(68.2)	
Black	192(6.0)	232(7.2)		317(15.0)	323(15.3)		198(25.7)	218(28.3)	
Other	500(15.6)	513(16.0)		151(7.1)	153(7.2)		24(3.1)	27(3.5)	
Grade			0.003			0.012			0.005
1	503(15.7)	477(14.9)		72(3.4)	72(3.4)		7(0.9)	11(1.4)	
2	1679(52.3)	1686(52.5)		740(35.0)	734(34.7)		108(14.0)	112(14.5)	
3	1029(32.0)	1048(32.6)		1305(61.6)	1311(61.9)		656(85.1)	648(84.0)	
T stage			-0.002			0.001			0.007
T1	1613(50.2)	1613(50.2)		453(21.4)	445(21.0)		61(7.9)	65(8.4)	
T2	1598(49.8)	1598(49.8)		1664(78.6)	1672(79.0)		710(92.1)	706(91.6)	
Chemotherapy			0.004			0.002			0.001
No	190(5.9)	174(5.4)		186(8.8)	181(8.5)		100(13.0)	100(13.0)	
Yes	3021(94.1)	3037(94.6)		1931(91.2)	1936(91.5)		671(87.0)	671(87.0)	
Examined lymph nodes			-0.001			0.009			-0.018
≤12	1546(48.1)	1503(46.8)		1174(55.5)	1154(54.5)		486(63.0)	483(62.6)	
>12	1665(51.9)	1708(53.2)		943(44.5)	963(45.5)		285(37.0)	288(37.4)	
Positive lymph nodes			-0.013			-0.007			-0.014
1	1650(51.4)	1682(52.4)		844(39.9)	862(40.7)		216(28.0)	243(31.5)	
2	1151(35.8)	1118(34.8)		753(35.6)	726(34.3)		246(31.9)	201(26.1)	
3	410(12.8)	411(12.8)		520(24.6)	529(25.0)		309(40.1)	327(42.4)	
ER			-0.002			-0.009			-0.008
Negative	176(5.5)	185(5.8)		677(32.0)	702(33.2)		515(66.8)	515(66.8)	
Positive	3035(94.5)	3026(94.2)		1440(68.0)	1415(66.8)		256(33.2)	256(33.2)	
PR			-0.006			-0.017			-0.030
Negative	398(12.4)	424(13.2)		966(45.6)	999(47.2)		611(79.2)	623(80.8)	
Positive	2813(87.6)	2787(86.8)		1151(54.4)	1118(52.8)		160(20.8)	148(19.2)	

USDW, unmarried/separated/divorced/widowed; Other, American Indian/AK Native, Asian/Pacific Islander; grade 1, well differentiated; grade 2, moderately differentiated; grade 3, poorly differentiated/undifferentiated; ALND, axillary lymph node dissection; ER, estrogen receptor; PR, progesterone receptor; HER2, human epidermal growth factor receptor 2; SMD, standardized mean difference.

### Benefits of Receiving PMRT in T1–2 Breast Cancer With 1–3 Positive Axillary Lymph Nodes Based on the PSM

**Figure 4:**
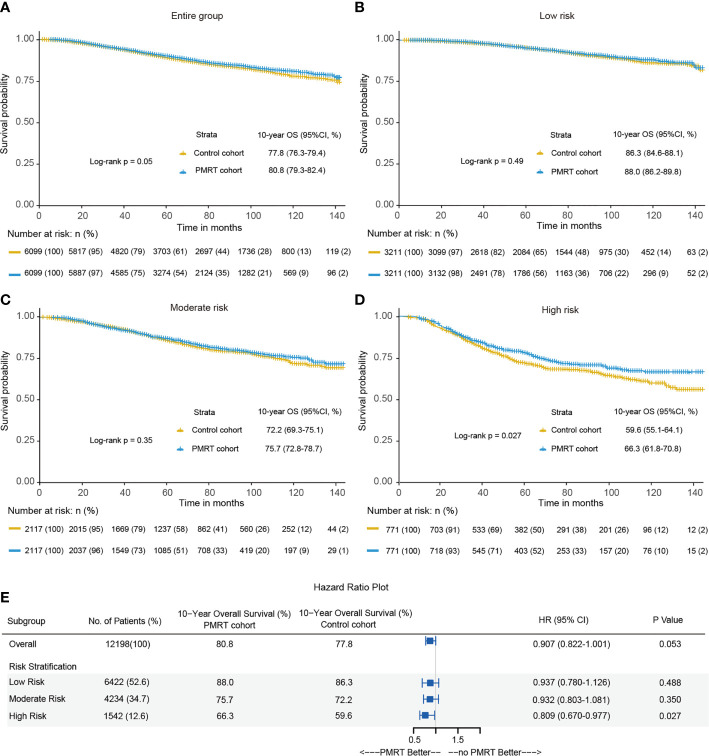
Kaplan-Meier curves of overall survival between the PMRT cohort and observation cohort for the entire group **(A)**, low-risk group **(B)**, moderate-risk group **(C)** and high-risk group **(D)** after PSM. **(E)** The forest plot for hazard ratio (HR) comparing 10-year overall survival between the control cohort and PMRT cohort in different risk groups after PSM.

All direct survival differences between the PMRT cohort and control cohort before the PSM were presented in **Supplementary Figure 2**. 10-year OS was 80.8% in the PMRT cohort and 77.8% in the control cohort after PSM (P = 0.05) (**Figure 4A**). PMRT improved the OS of patients in the high-risk group but did not better OS among those in the low-risk and moderate-risk groups. In the low-risk group, 10-year OS was nearly equivalent (P = 0.49), with 88.0% in the PMRT cohort and 86.3% in the control cohort (**Figure 4B**). In the moderate-risk group, 10-year OS rates of PMRT cohort and control cohort were 75.7% and 72.2%, respectively (P = 0.35) (**Figure 4C**). In the high-risk group, PMRT can significantly improve 10-year OS, with 66.3% in the PMRT cohort and 59.6% in the control cohort (**Figure 4D**). This study found that PMRT can significantly improve the OS of T1–2 breast cancer with 1–3 positive lymph nodes in the high-risk group (**Figure 4E**).

## Discussion

Based on the data of 18607 T1–2 breast cancer with 1–3 positive axillary lymph nodes from the real-world, we determined its independent prognostic factors, developed a prognostic stratification model that can predict individual prognosis with favorable accuracy and discrimination, and applied the model to stratify the entire cohort into different risk groups to identify the optimal candidates benefiting from PMRT. In the entire cohort, PMRT did not improve 10-year OS of T1–2 breast cancer with 1–3 positive axillary lymph nodes significantly. In the low-risk group and moderate-risk group, 10-year OS rates of the PMRT cohort and control cohort were not significantly different, while PMRT can significantly improve the 10-year OS rate in the high-risk group. The innovation and advantage of this research lies in: (a) conducting the study based on a large sample size; (b) building up the first prognostic stratification nomogram specially for T1–2 breast cancer with 1–3 positive axillary lymph nodes based on multi-ethnic population; (c) validating excellent performance of risk stratification of nomogram by using Kaplan-Meier method and decision curve analysis; (d) applying PSM to balance the baseline characteristics between the PMRT cohort and control cohort in prognostic stratification groups to minimize the confounding factors of independent features; (e) finding that PMRT could effectively improve the OS of patients in the high-risk group after PSM, with 10-year OS absolute improvement by 6.7%.

In this study, marital status, age, race, grade, T stage, chemotherapy status, examined nodes, number of positive axillary lymph nodes, ER status, and PR status were independent prognostic factors for T1–2 breast cancer with 1–3 positive axillary lymph nodes. Consistent with the previous research results, grade 3, T2, no chemotherapy, fewer examined lymph nodes (4–13), more positive axillary lymph nodes, ER-negative, and PR-negative were independent risk factors for prognosis of T1–2 breast cancer with 1–3 positive axillary lymph nodes (11–24, 37). Also, our study indicated that patients with USDW (unmarried/separated/divorced/widowed) in marital status and black in race often have a high prognostic risk. Although such findings have not been reported in T1–2 breast cancer with 1–3 positive axillary lymph nodes, studies both breast cancer and metastatic breast cancer have reported similar results (38, 39). Interestingly, our analysis demonstrated that the 60–69 group was an independent risk factor for OS more than the 20-39 group, which seemed partly different from those studies reported that 40 years old or younger was a significant risk factor of T1–2 breast cancer with 1–3 positive axillary lymph nodes (12, 18–24). Consistent with the findings of Zhang et al. (40), this is mainly due to the fact that people aged 60-69 have more other serious diseases (such as diseases of heart, and chronic obstructive pulmonary disease and allied cond), and had a higher incidence of all-cause mortality compared with patients aged 20-39.

Considering that T1–2 breast cancer with 1–3 positive axillary lymph nodes had several independent prognostic factors, we incorporated all factors into the prognostic stratification model. The prognostic model can stratify the prognosis of patients into three risk groups. Our analysis proposed that patients in the high-risk group may be the potential population that could benefit from PMRT. Patients from the high-risk group have more independent risk factors and possibly higher all-cause mortality than those in the low-risk and moderate-risk groups. The escalation of treatment provided patients with better local control and prolongs survival to patients with higher death risk. Recently, several studies have tried to identify potential candidates who could benefit from PMRT in T1–2 breast cancer with 1–3 positive axillary lymph nodes. Chen et al. and Dai et al. reported that PMRT could only improve the prognosis of patients with three positive axillary lymph nodes (41, 42). A study conducted in the University of Chicago revealed that PMRT could reduce the all-cause mortality of patients with two positive lymph nodes and tumors 2–5 cm in size or three positive nodes by 14% (26). A clinical study in Korea revealed that PMRT could significantly better the prognosis for patients with an intermediate ratio of positive lymph nodes to total nodes dissected (23). Almost all these studies only took tumor burden (tumor size and number of positive lymph nodes) into consideration and did not take other critical factors seriously. Our study comprehensively incorporated all independent factors into prognostic nomogram and compared the net benefit between the risk stratification nomogram-assisted decision and single factor-assisted decisions. Notably, the net benefit of decisions based on the model and its risk stratification were significantly higher than those of the number of positive lymph nodes and grade proposed by previous researchers (41, 42). Although a multi-center study in China had established the first risk stratification nomogram for T1-2 breast cancer with 1-3 positive lymph nodes, it only included the Chinese population. Due to differences of race and other factors, the model applied in other races may have great limitations (43).

The current study built up the novel risk stratification model of T1–2 breast cancer with 1–3 positive axillary lymph nodes, which could accurately stratify the prognosis of patients into different risk groups and determine which patients were optimal candidates who may benefit from PMRT. In principle, our nomogram could identify whether T1–2 breast cancer with 1–3 positive axillary lymph nodes were potential population benefiting from PMRT. Although our study had many advantages, there were still several limitations: (a) the SEER database lacked detailed information on chemotherapy, endocrine therapy, and the dose, area, and complications of radiotherapy, so our study may have confounding factors; (b) there was no data on recurrence after surgery in the database, so we cannot identify the impact of PMRT on recurrence; (c) this study was limited by its retrospective design, but the PSM method was performed to reduce the confounding factors of independent features; (d) our prognostic stratification model had not been verified by other centers or databases (e.g., National Cancer Database, SEER-Medicare, etc.).

## Conclusion

This study constructed a novel prognostic stratification model for T1–2 breast cancer with 1–3 positive axillary lymph nodes, which could help determine the potential population who could benefit from PMRT. The prognostic stratification model was expected to promote individual treatment of PMRT for T1–2 breast cancer with 1–3 positive axillary lymph nodes so that patients would benefit most from PMRT.

## Data Availability Statement

Publicly available datasets were analyzed in this study. This data can be found here: https://seer.cancer.gov/data/.

## Ethics Statement

This study was approved by the ethics committee of the local hospital (Xijing hospital), and because it was a retrospective study, permission for the exemption of informed consent was obtained.

## Author Contributions

RL and MS designed the study. NH, JZ, LuY, and YW wrote the primary manuscript. NH, ZW, MZ, LiY, and GH extracted the data from the SEER database. NH, JW, YDW, BD, and LG performed the statistical analysis. All authors read and approved the final manuscript.

## Funding

This study was supported by the National Natural Science Foundation of China (Nos. 81572917).

## Conflict of Interest

The authors declare that the research was conducted in the absence of any commercial or financial relationships that could be construed as a potential conflict of interest.
