# Supplementary material for: A Prognostic Risk Stratification Model to Identify Potential Population Benefiting From Postmastectomy Radiotherapy in T1–2 Breast Cancer With 1–3 Positive Axillary Lymph Nodes
Source: Front Oncol. 2021 Apr 19;11:640268. doi: 10.3389/fonc.2021.640268 (PMC8089395; doi:10.3389/fonc.2021.640268)
Supplement: Supplementary file 1 [file DataSheet_1.pdf]

Supplementary Fig. 1. Identification of the optimal cut-offs for T1-2N1M0 breast cancer in non-PMRT cohort using X-tile software.

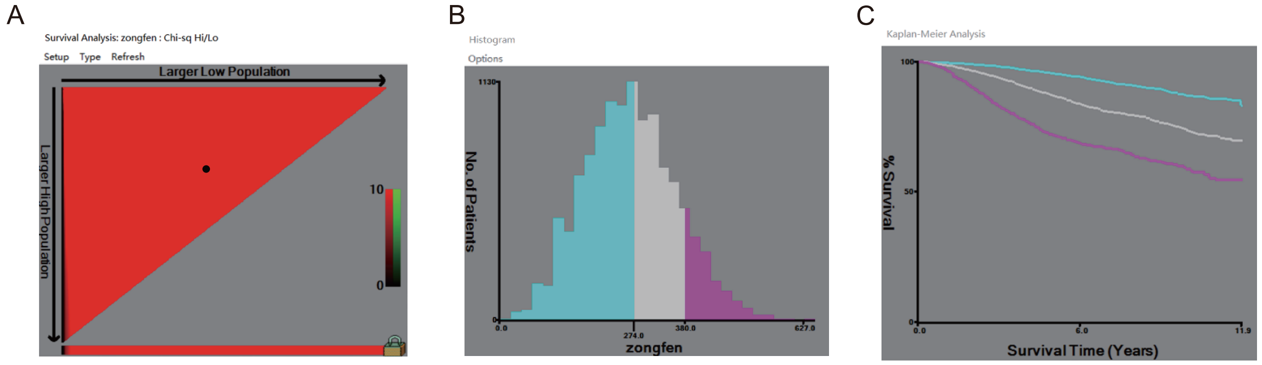

Supplementary Fig. 2. Kaplan-Meier curves of overall survival between the PMRT cohort and control cohort for the entire group (A), low-risk group (B), moderate-risk group (C) and high-risk group (D) before PSM.

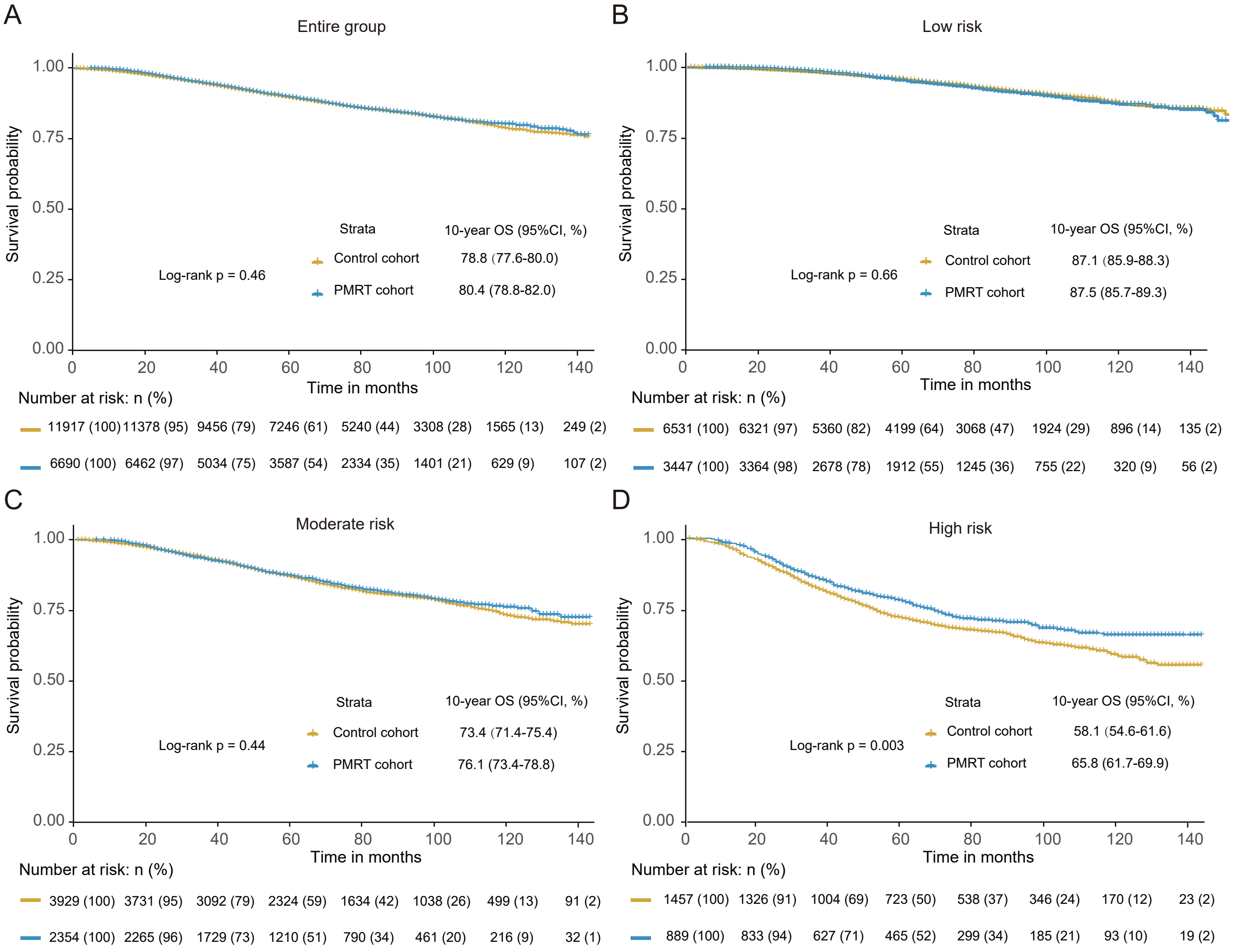

Supplementary Table 1 Demographic and clinicopathologic features of patients between the control cohort and validation cohort in T1-2 breast cancer with 1-3 positive lymph nodes.

Characteristic		Control cohort (11917)	Validation cohort (1110)	P
Marital status				0.082
	Married	7761(65.1)	694(62.5)	
	USDW	4156(34.9)	416(37.5)	
Age (years)				0.060
	20-39	1223(10.3)	130(11.7)	
	40-49	3465(29.1)	333(30.0)	
	50-59	3908(32.8)	377(34.0)	
	60-69	3321(27.9)	270(24.3)	
Race				0.429
	White	9333(78.3)	854(76.9)	
	Black	1291(10.8)	134(12.1)	
	Other	1293(10.9)	122(11.0)	
Histology				0.009
	Infiltrating duct cancer	10871(91.2)	1038(93.5)	
	Infiltrating lobular carcinoma	1046(8.8)	72(6.5)	
Grade				0.041
	1	1578(13.2)	131(11.8)	
	2	5172(43.4)	455(41.0)	
	3	5167(43.4)	524(47.2)	
Laterality				0.959
	Left	6024(50.5)	562(50.6)	
	Right	5893(49.5)	548(49.4)	
T stage				0.956
	T1	5346(44.9)	497(44.8)	
	T2	6571(55.1)	613(55.2)	
Chemotherapy				0.348
	No	3259(27.3)	289(26.0)	
	Yes	8658(72.7)	821(74.0)	
ALND				<0.001
	No	4289(36.0)	266(24.0)	
	Yes	7628(64.0)	844(76.0)	
Examined lymph nodes				0.836
	Median (IQR)	12(8-17)	12(8-17)	
Positive lymph nodes				0.381
	1	6701(56.2)	607(54.7)	
	2	3562(29.9)	333(30.0)	
	3	1654(13.9)	170(15.3)	
ER				<0.001

	Negative	2350(19.7)	298(26.8)	
	Positive	9567(80.3)	812(73.2)	
PR				<0.001
	Negative	3526(29.6)	426(38.4)	
	Positive	8391(70.4)	684(61.6)	

USDW, unmarried/separated/divorced/widowed; Other, American Indian/AK Native, Asian/Pacific Islander; grade 1, well differentiated; grade 2, moderately differentiated; grade 3, poorly differentiated / undifferentiated; ALND, axillary lymph node dissection; ER, estrogen receptor; PR, progesterone receptor; HER2, human epidermal growth factor receptor 2.

Supplementary Table 2 The baseline characteristics of the patients with PMRT or observation in each risk group before the PSM.

Characteristic	Low-risk group			Moderate-risk group			High-risk group		
	Control cohort (6531)	PMRT cohort (3447)	SMD	Control cohort (3929)	PMRT cohort (2354)	SMD	Control cohort (1457)	PMRT cohort (889)	SMD
Marital Status			0.029			0.009			0.007
Married	5117(78.3)	2801(81.3)		2162(55.0)	1317(55.9)		482(33.1)	300(33.7)	
USDW	1414(21.7)	646(18.7)		1767(45.0)	1037(44.1)		975(66.9)	589(66.3)	
Age (years)			**-0.22 2**			**-0.29 2**			**-0.29 6**
20-39	707(10.8)	549(15.9)		415(10.6)	421(17.9)		101(6.9)	123(13.8)	
40-49	2593(39.7)	1577(45.7)		773(19.7)	562(23.9)		99(6.8)	101(11.4)	
50-59	2343(35.9)	1058(30.7)		1211(30.8)	698(29.7)		354(24.3)	214(24.1)	
60-69	888(13.6)	263(7.6)		1530(38.9)	673(28.6)		903(62.0)	451(50.7)	
Race			0.037			0.020			0.044
White	5166(79.1)	2643(76.7)		3106(79.1)	1830(77.7)		1061(72.8)	607(68.3)	
Black	402(6.2)	251(7.3)		553(14.1)	347(14.7)		336(23.1)	247(27.8)	
Other	963(14.7)	553(16.0)		270(6.9)	177(7.5)		60(4.1)	35(3.9)	
Grade			0.096			**0.101**			0.055
1	1319(20.2)	496(14.4)		236(6.0)	75(3.2)		23(1.6)	11(1.2)	
2	3355(51.4)	1842(53.4)		1534(39.0)	814(34.6)		283(19.4)	130(14.6)	
3	1857(28.4)	1109(32.2)		2159(55.0)	1465(62.2)		1151(79.0)	748(84.1)	
T stage			**0.134**			0.088			0.024
T1	4076(62.4)	1688(49.0)		1126(28.7)	467(19.8)		144(9.9)	67(7.5)	
T2	2455(37.6)	1759(51.0)		2803(71.3)	1887(80.2)		1313(90.1)	822(92.5)	
Chemotherapy			**0.162**			**0.246**			**0.301**
No	1386(21.2)	174(5.0)		1269(32.3)	181(7.7)		604(41.5)	101(11.4)	
Yes	5145(78.8)	3273(95.0)		2660(67.7)	2173(92.3)		853(58.5)	788(88.6)	
Examined lymph nodes			-0.03 4			-0.01 2			0.001
1-12	2892(44.3)	1644(47.7)		2152(54.8)	1317(55.9)		921(63.2)	561(63.1)	
>13	3639(55.7)	1803(52.3)		1777(45.2)	1037(44.1)		536(36.8)	328(36.9)	
Positive lymph nodes			**0.235**			**0.256**			**0.239**
1	4160(63.7)	1694(49.1)		1994(50.8)	875(37.2)		547(37.5)	246(27.7)	
2	1881(28.8)	1186(34.4)		1249(31.8)	785(33.3)		432(29.6)	227(25.5)	
3	490(7.5)	567(16.4)		686(17.5)	694(29.5)		478(32.8)	416(46.8)	
ER			0.002			-0.01 7			-0.07 5
Negative	367(5.6)	187(5.4)		1138(29.0)	721(30.6)		845(58.0)	582(65.5)	
Positive	6164(94.4)	3260(94.6)		2791(71.0)	1633(69.4)		612(42.0)	307(34.5)	
PR			-0.00			-0.02			-0.06

			1			2			1
Negative	824(12.6)	439(12.7)		1647(41.9)	1038(44.1)		1055(72.4)	698(78.5)	
Positive	5707(87.4)	3008(87.3)		2282(58.1)	1316(55.9)		402(27.6)	191(21.5)	

USDW, unmarried/separated/divorced/widowed; Other, American Indian/AK Native, Asian/Pacific Islander; grade 1, well differentiated; grade 2, moderately differentiated; grade 3, poorly differentiated / undifferentiated; ER, estrogen receptor; PR, progesterone receptor; HER2, human epidermal growth factor receptor 2. SMD, standardized mean difference > 0.10 was marked in bold.
